# Bacterial Abilities and Adaptation Toward the Rhizosphere Colonization

**DOI:** 10.3389/fmicb.2016.01341

**Published:** 2016-08-25

**Authors:** Lucas D. Lopes, Michele de Cássia Pereira e Silva, Fernando D. Andreote

**Affiliations:** Soil Microbiology Lab, Department of Soil Science, “Luiz de Queiroz” College of Agriculture, University of São PauloPiracicaba, Brazil

**Keywords:** microbiome, sugarcane, metagenome prediction, plant selection, horizontal gene transfer

## Abstract

The rhizosphere harbors one of the most complex, diverse, and active plant-associated microbial communities. This community can be recruited by the plant host to either supply it with nutrients or to help in the survival under stressful conditions. Although selection for the rhizosphere community is evident, the specific bacterial traits that make them able to colonize this environment are still poorly understood. Thus, here we used a combination of community level physiological profile (CLPP) analysis and 16S rRNA gene quantification and sequencing (coupled with *in silico* analysis and metagenome prediction), to get insights on bacterial features and processes involved in rhizosphere colonization of sugarcane. CLPP revealed a higher metabolic activity in the rhizosphere compared to bulk soil, and suggested that D-galacturonic acid plays a role in bacterial selection by the plant roots (supported by results of metagenome prediction). Quantification of the 16S rRNA gene confirmed the higher abundance of bacteria in the rhizosphere. Sequence analysis showed that of the 252 classified families sampled, 24 were significantly more abundant in the bulk soil and 29 were more abundant in the rhizosphere. Furthermore, metagenomes predicted from the 16S rRNA gene sequences revealed a significant higher abundance of predicted genes associated with biofilm formation and with horizontal gene transfer (HGT) processes. In sum, this study identified major bacterial groups and their potential abilities to occupy the sugarcane rhizosphere, and indicated that polygalacturonase activity and HGT events may be important features for rhizosphere colonization.

## Introduction

Of the various niches that comprise the plant microbiome – i.e., phyllosphere, spermosphere, rhizosphere, internal tissues (Turner et al., 2013; Andreote et al., 2014)- the most complex, diverse, and active microbial community is located in the rhizosphere, the soil layer immediately influenced by the rhizodeposition (Prashar et al., 2014). In the rhizosphere, there is a selection effect imposed by the physicochemical changes created in the roots’ surroundings that shape the microbial composition. This selection can happen both indirectly, favoring the growth of opportunistic microbes adapted to specific chemical conditions, or by the active recruitment of microbes that will support plant development, support plant growth/nutrition, and/or enhancing the plant’s ability to resist biotic and abiotic stressors (Bulgarelli et al., 2013; Philippot et al., 2013; Mendes R. et al., 2014). These microbial partners are understood to receive labile forms of carbon and a continuing supply of nutrients from the plants in exchange to their services (Bais et al., 2006).

Bulk soil, i.e., the zone of soil not under the influence of rhizodeposition, houses a huge microbial diversity and is considered the ultimate reservoir of microbes available for plant colonization, from which microbes are selected to the rhizosphere microbiome (Uroz et al., 2010; Peiffer et al., 2013; Mendes L.W. et al., 2014). However, the set of features required for bacteria to efficiently colonize the rhizosphere is yet to be properly described. For instance, studies have shown the importance of motility (de Weger et al., 1987) and lipopolysaccharide (LPS) production (de Weger et al., 1989) for the colonization of potato roots by a *Pseudomonas fluorescens* strain. The capacity to form biofilm was shown to be related to the rhizosphere colonization in *Bacillus amyloliquefaciens* and rhizobia species (Rinaudi and Giordano, 2010; Tan et al., 2013), but not in *P. fluorescens* (Barahona et al., 2010). Fast growth rate was suggested to be important for rhizosphere colonization in *Pseudomonas* spp. and *B. amyloliquefaciens* (Simons et al., 1996; Tan et al., 2013). In addition, the chemotaxis exerted by some organic acids was significant for the colonization of tomato roots by *B. amyloliquefaciens* (Tan et al., 2013). Another less explored ecological aspect possibly involved in the rhizosphere colonization by soil bacteria is the suitability of bacteria for horizontal gene transfer (HGT) processes, which could hypothetically support the rapid adaptation of bacteria in the face of environmental shifts. There are increasing indications that the rhizosphere could be a hot spot of HGT events, for example, by the increase of the transference of conjugative plasmids between rhizosphere inhabitants (Lilley et al., 1994; Pukall et al., 1996; Van Elsas et al., 1998). Although some studies have identified traits related to the colonization of specific bacterial species, there is still a gap in the knowledge of key features involved in rhizosphere colonization by the soil microbiome.

Thus, the aim of the present study was to identify key bacterial traits for rhizosphere colonization using sugarcane as a model system. We studied the bacterial communities and assessed shifts in the taxonomic and functional profiles in bulk soil in comparison to the rhizosphere. Analyzes were performed using a combination of bacterial quantification, metabolic capacity to degrade carbon sources, high-throughput sequencing and metagenome prediction. Using this approach we gained a better understanding about the taxonomic and ecological relationships that microorganisms establish in the sugarcane rhizosphere, as well as of some of the characteristics needed for bacterial communities to colonize this soil habitat, including polygalacturonase activity and the possible importance of HGT in the rhizosphere.

## Materials and Methods

### Bulk Soil and Rhizosphere Sampling

Bulk soil and rhizosphere were sampled in a sugarcane cultivation field (cultivar SP-3250) located at ESALQ/USP (Piracicaba, Brazil). The sugarcane crop is being used in this field for 10 years under a green harvest management. Plants sampled in this study were at 9-months of cultivation (average height of 2.0 m) and did not show evidence for pest attack, disease, or nutritional deficiency. Bulk soil samples were made of soils free of roots, collected in the interline area of planting, at the layer of 0–10 cm. Rhizosphere samples were obtained by separating soil from plants roots (similar soil depth), focusing on a soil layer not thicker than 2 mm from the roots surface. Six biological replicates were used, each comprising of a single plant from which bulk soil and rhizosphere samples were collected, generating a total of 12 samples.

### DNA Extraction

Total DNA was extracted from each sample using a MoBio Power Soil DNA Isolation Kit (Mobio, USA) according to the manufacturer’s instructions. The resulting DNA was checked for integrity by electrophoresis in a 1% agarose gel, stained with ethidium bromide and visualized under UV light, and stored at -20°C.

### Quantification of Bacterial Community

The 16S rRNA gene copy numbers in bulk soil and rhizosphere were assessed to investigate whether these two habitats harbor significantly different bacterial abundances. Real-time PCR amplification was performed using P1/P2 primers (Muyzer et al., 1993) in a reaction with 12.5 μL of Sybr Green (1X), 10 mM of each primer, and 1 μL of template DNA for a total volume of 25 μL. The amplification was conducted using a StepOne Real-Time System (Applied Biosystems) under the following conditions: 1 cycle of 95°C for 3 min, 35 cycles of 94°C for 30 s, 55°C for 30 s and 72°C for 30 s, followed by a melting curve analysis. For the standard curve, soil-derived amplicons of the 16S rRNA gene were diluted from 10^-1^ to 10^-8^ and quantified. These dilutions were submitted to amplifications under the same conditions described above. In addition, the efficiency of the reaction was calculated and was 101.5%, between the acceptable values of 90 and 110%, indicating the absence of PCR inhibitors.

### Sequencing of the 16S rRNA Gene

We performed a sequence-based analysis using the Illumina MiSeq platform and the Nextera XT index kit for library preparation (Illumina, USA), targeting the V3-V4 region of the 16S rRNA gene. A nested protocol was performed. In the first reaction the whole gene was amplified using the universal primers 27F (Lane, 1991) and 1387R (Marchesi et al., 1998). This first reaction contained 1 μL of template, 2 μL of dNTPs (2.5 mM), 3.75 μL of MgCl_2_ (25 mM), 2.5 μL of Taq buffer (10X), 0.1 μL of each primer (100 mM) and 0.3 μL of Taq polymerase (5U/μL) for a total volume of 25 μL. The thermal cycles consisted of 1 cycle of 94°C for 4 min, 25 cycles of 94°C (30 s), 63°C (1 min) and 72°C (1 min), ending with 1 cycle of 72°C for 10 min. In the second reaction, primers S-D-Bact-0341-b-S-17/S-D-Bact-0785-a-A-21 (Klindworth et al., 2013) were used (coupled with Illumina adapters), which cover the hypervariable regions V3-V4 of the 16S rRNA gene. This reaction was conducted using 1 μL of the amplicons produced in the first reaction, 2 μL of dNTPs (2.5 mM), 3 μL of MgCl2 (25 mM), 3 μL of Taq Buffer (10X), 0.1 μL of each primer and 0.3 μL of Taq DNA polymerase for a total volume of 25 μL. The amplification was conducted following the thermal cycles: an initial denaturation cycle of 95° for 3 min, 30 cycles of 95°C for 45 s (denaturation), 57°C for 1 min and 45 s (annealing) and 72°C for 1 min (extension), followed by a final extension cycle of 72°C for 4 min. The primers sequences are found in Supplementary Table S1. Nextera indices and sequencing adapters were ligated to amplicons from the second reaction, and submitted for paired-end sequencing on the Illumina MiSeq platform. The reads were deposited at the SRA database (NCBI), BioProject PRJNA319762, and the BioSample accession numbers are: SAMN04904299 (B1); SAMN04904300 (B2); SAMN04904301 (B3); SAMN04904302 (B4); SAMN04904303 (B5); SAMN04904304 (B6); SAMN04904305 (R1); SAMN04904306 (R2); SAMN04904307 (R3); SAMN04904308 (R4); SAMN04904309 (R5); and SAMN04904310 (R6) (R = Rhizosphere and B = Bulk Soil samples).

### Next Generation Sequence Analysis

The sequenced paired-end reads were separated by sample and analyzed using the QIIME software pipeline (Caporaso et al., 2010). Quality control analyses were performed to eliminate low quality reads, short reads, chimeric sequences, and to trim the low quality 3′ region of individual reads in order to achieve a minimum quality of Q28 (Phred scale). The UCLUST algorithm was used to cluster the reads in operational taxonomic units (OTUs) with a 97% cutoff, and to assign the taxonomy using the Greengenes database (version gg_13_8_otus) with a minimum fraction of 0.51. The reads were then aligned using the PyNAST algorithm and filtered. An OTU table was generated, the singletons were excluded and the OTU table was rarified (170,000 sequences) to avoid bias related to different number of reads in the samples. Richness and diversity indices were calculated, and the OTU table was exported to STAMP (Parks et al., 2014) for statistical analyzes.

In addition to the amplicon-based analysis, we also used a metagenome prediction approach to infer probable functions performed by the bacterial communities, which is more informative than a purely taxonomic community structure approach. For this, we used the PICRUSt software package, which uses 16S rRNA libraries to make a predicted reconstruction of the metagenome (Langille et al., 2013). The PICRUSt software uses an evolutionary approach to handle the OTUs that match with unavailable genomes by using their sequenced relatives as a reference for the prediction. This means that there is some uncertainty in the genome prediction, as all microbiome sequencing produces some OTUs that match with unavailable genomes and different strains of the same species/OTUs have some distinct gene content. The nearest sequenced taxon index (NSTI) value show the level of uncertainty of the metagenome prediction, increasing the accuracy of the prediction the smaller are its values. First, a new OTU table was created using a closed-reference picking OTU protocol against the Greengenes database (version gg_13_5_otus) at 97% identity, which was then normalized by dividing the abundances of each OTU by known or predicted 16S rRNA gene copy number abundances. The normalized data was then submitted to metagenome prediction and categorized by function using KEGG level 2 Gene Ontology (GO) terms for classification. The NSTI was used to quantify the availability of nearby genome representatives in the samples. The prediction tables (the raw gene prediction table and the classified table with the GO terms) were then exported to STAMP software (Parks et al., 2014) for statistical analysis.

### Community Level Physiological Profile (CLPP) Analysis

To better characterize the functional profile of the microbial communities in our samples, we performed a CLPP analysis using BIOLOG Ecoplates (Biolog Inc., USA), which contains 31 different carbon sources. For this analysis, 10 g of either soil or rhizosphere samples were weighed, and a soil suspension was obtained by shaking the samples in 90 mL of saline solution (0.1 M NaCl) for 30 min. This suspension was then centrifuged for 30 min at 250 rpm, the supernatant was diluted to 10^-3^ and 150 μl was used to inoculate the Ecoplates. Plates were incubated at 25°C and read at the 590 nM wavelength in 24 h intervals for a total of 168 h. Absorbance readings were corrected using blank samples as controls.

### Statistical Analysis

Results of qPCR were analyzed using the PAST software (Hammer et al., 2001), where ANOVA and Tukey pairwise comparison tests were performed to assess differences in rhizosphere and bulk soil samples.

The OTU table of the 16S rRNA gene sequencing was exported and analyzed in PRIMER-6 software (Clarke and Gorley, 2006) where a Non-metric Multidimensional Scaling (NMDS) as well as an Analysis of Similarity (ANOSIM) were performed using the Bray–Curtis dissimilarity matrix with the aim of detecting differences in bacterial community structure between the 2 habitats studied (rhizosphere × bulk soil). These identified differences were further explored using the STAMP software (Parks et al., 2014), where bacterial groups with significant differences were identified using the Welch’s t-test (Parks et al., 2014). The same approach was used to identify significant differences in the predicted metagenomes of rhizosphere and bulk soil samples. The Benjamini–Hochberg *P*-value correction was utilized to avoid type 1 and 2 errors. In addition, a Bonferroni *P*-value correction was applied to the raw predicted genes table to account for potential bias related to PICRUSt predictions and statistical analysis, as this correction is even more conservative in detecting significant differences.

The PAST software was used to perform an ANOVA and Tukey test for each variable (C-sources) of the CLPP results. Alternatively, the results were exported to the CANOCO 4.5 software, where a biplot Principal Component Analysis (PCA) was performed to check the differences in the metabolic profiles of the microbial communities of rhizosphere and bulk soil, as well as to detect any correlation with the C-sources (ter Braak and Smilauer, 2002). An ANOSIM was also performed to validate the significance of these differences using the PRIMER-6 software package (Clarke and Gorley, 2006). The niche of a given community, bulk soil or rhizosphere, was calculated according to Salles et al. (2009). This analysis is based on the performance of the total community in each of the carbon sources, calculated as the sum of the best performances on each source present on the environment where that community is functioning.

## Results

### Distinctions of Bacterial Abundance and Community Structure between Rhizosphere and Bulk Soil

**FIGURE 1:**
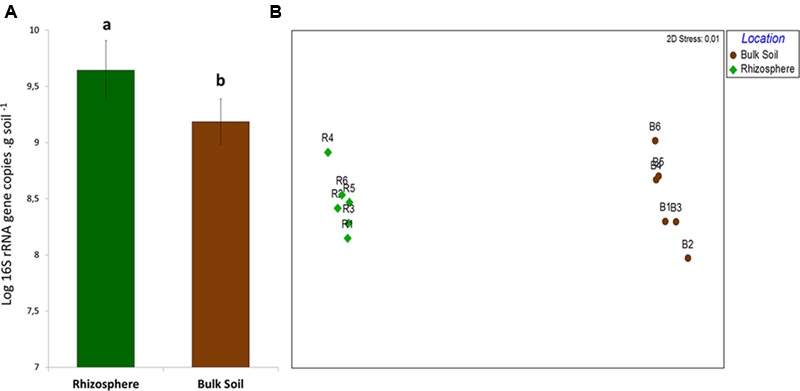
**(A)** Quantification of 16S rRNA gene in samples of bulk soil and rhizosphere. Different letters means significant differences according to the pairwise comparison test of Tukey (*P* < 0.05). **(B)** Non-Metric Multidiomensional Scaling (NMDS) comparing the structure of bacterial community in bulk soil and rhizosphere on the basis of the OTU table.

Quantification of total bacterial communities in bulk soil and rhizosphere samples revealed significant differences in bacterial abundance (*P* < 0.05). The average bacterial abundance in rhizosphere samples was 4.96 × 10^9^ rRNA gene copies per gram of soil, while bulk soil samples contained an average of 1.67 × 10^9^ (**Figure 1A**). This distinction was complemented by the differential structuring of bacterial communities in each niche, as indicated by the results from partial sequencing of the 16S rRNA gene. After trimming sequences of low quality and rarification of sequences per sample, a total of 2,040,000 sequences – 1,020,000 from each rhizosphere and bulk soil samples – were used in the analysis.

Non-metric Multidimensional Scaling results indicated the distinct structure of bacterial communities in rhizosphere and bulk soil samples – confirmed by ANOSIM (*P* = 0.008/*R* = 0.82). The two groups of samples were separated in the first axis, and replicates grouped together, clearly indicating the differential selection exerted by each of these niches upon the bacterial community (**Figure 1B**). Although there was a clear difference in β-diversity, no differences concerning the α-diversity estimators (*P* < 0.05) were observed between the niches (**Table 1**).

**Table 1:** The output of sequences analysis after quality filtering per sample, before and after the OTU table rarefaction.

		Before rarefaction	Post rarefaction on OTU table		
Location	Samples	N° of Reads	N° of Reads	Shannon index	Chao 1 index
Rhizosphere	R1	230,123	170,000	11.9991	93,359
	R2	271,867	170,000	12.0379	91,204
	R3	216,554	170,000	12.1031	93,485
	R4	243,438	170,000	12.2418	92,860
	R5	211,310	170,000	11.9151	85,191
	R6	172,230	170,000	11.8505	85,779

	Total	1,345,522	1,020,000	μ = 12.0246 *SD* = 0.1389	μ = 89,804 *SD* = 4,050.56

Bulk Soil	B1	213,884	170,000	11.9015	90,660
	B2	277,195	170,000	12.1156	95,930
	B3	271,546	170,000	11.9811	91,201
	B4	304,783	170,000	12.0649	92,421
	B5	201,595	170,000	11.9613	90,310
	B6	280,574	170,000	11.9765	96,428

	Total	1,549,577	1,020,000	μ = 12.0002 *SD* = 0.0770	μ = 93,350 *SD* = 2,734.53

*Calculation of Shannon diversity index, Chao 1 species richness index and their average (μ) and standard deviation (SD).*

**FIGURE 2:**
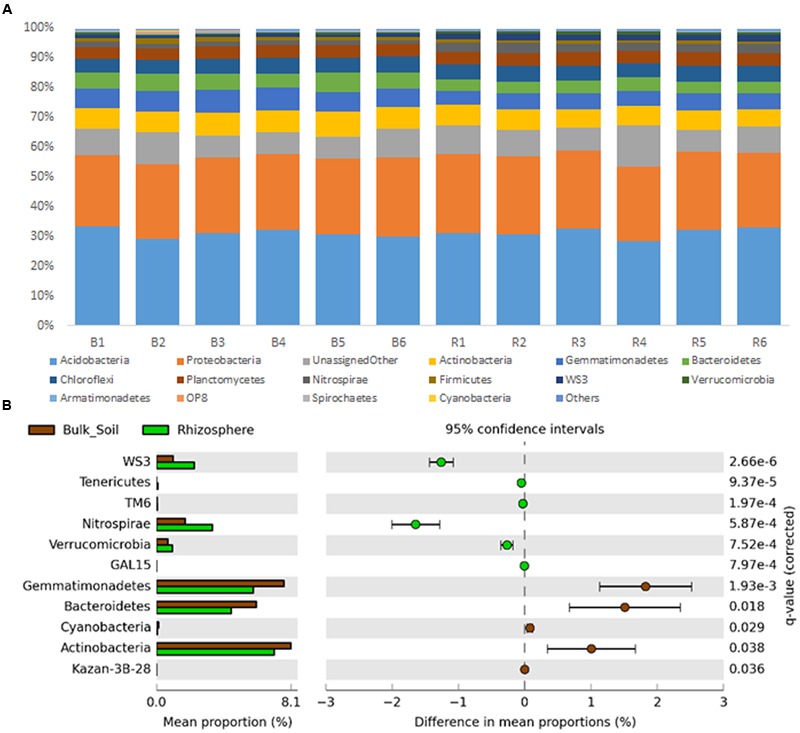
**(A)** Taxonomic classification of OTUs (at level of phyla) based on Greengenes database using the QIIME software. **(B)** Statistical comparison (Welch’s *t*-test) between the phyla abundance on rhizosphere and bulk soil using the Benjamini–Hochberg *P*-value correction (*P* < 0.05).

**FIGURE 3:**
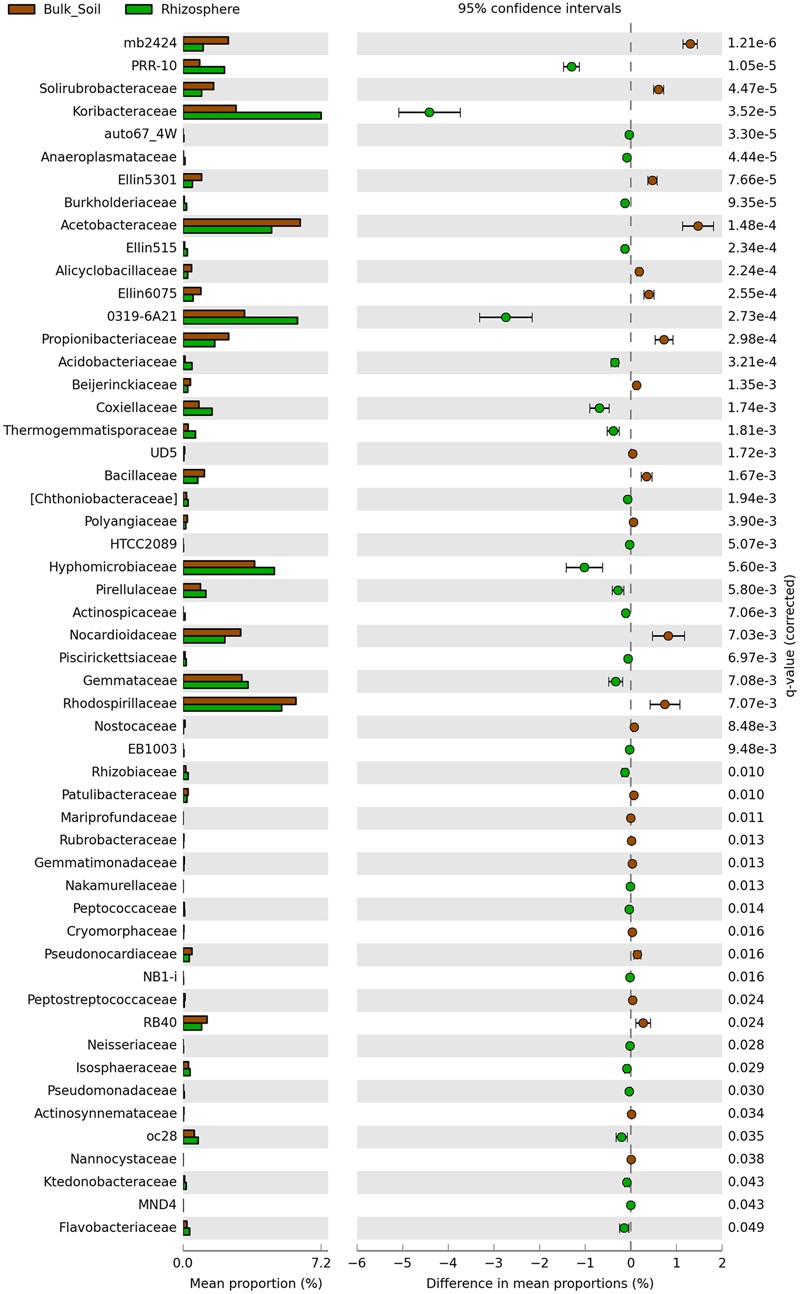
**Statistical comparison (Welch’s *t*-test) between the families abundance on rhizosphere and bulk soil using the Benjamini-Hochberg *P*-value correction (*P* < 0.05)**.

Sequences were classified into 24 known and 31 candidate bacterial phyla, with prevalence of sequences affiliated to Acidobacteria and Proteobacteria in both communities (**Figure 2A**). Divergences in the relative abundance of phyla Actinobacteria, Bacteroidetes, Cyanobacteria and Gemmatimonadetes were observed, which were more abundant in the bulk soil (*P* < 0.01). On the other hand, Verrucomicrobia, Nitrospirae and Tenericutes were more abundant in the rhizosphere (*P* < 0.01) (**Figure 2B**). Higher divergences were observed at family level (**Figure 3**), from which 24 were significantly more abundant in the bulk soil, and 29 were more abundant in the rhizosphere (*P* < 0.05).

### Metabolic Profiles of Rhizosphere and Bulk Soil Microbial Communities

**FIGURE 4:**
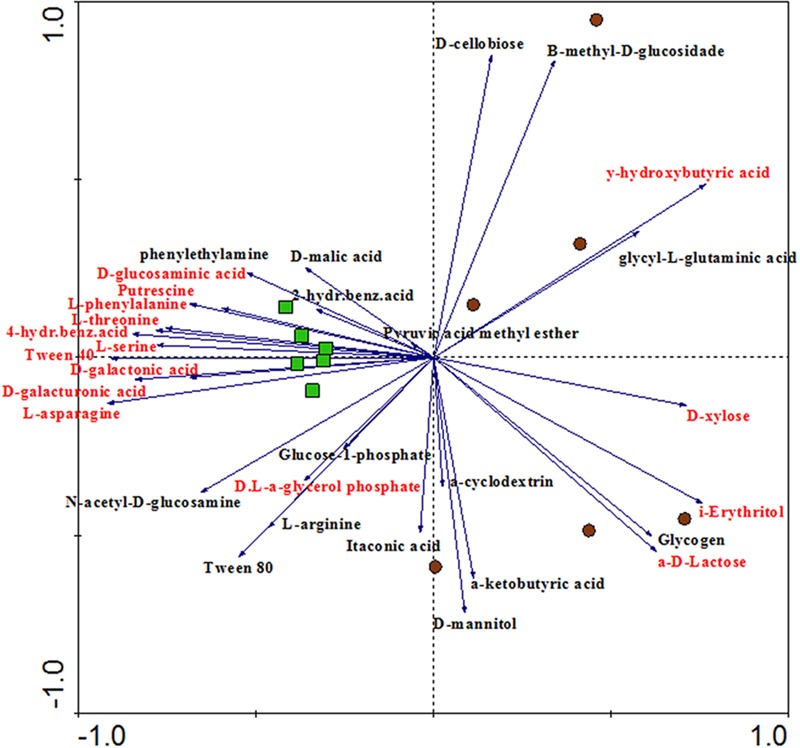
**Principal component analysis (PCA) showing the ordination of rhizosphere and bulk soil samples according to the oxidation of C-sources in the CLPP analysis.** Variables (C-sources) colored in red means significantly different between rhizosphere and bulk soil in the Tukey test (*P* < 0.05). Samples represented by green squares are from rhizosphere and samples represented by brown circles are from bulk soil (PC1 = 32.5%, PC2 = 21.3%).

CLPP analysis indicated differential metabolic patterns of the microbial communities found in bulk soil and rhizosphere. A broad view of such patterns was obtained with a PCA, which revealed clear distinctions between the carbon degradation profiles of the communities from each niche, supported by ANOSIM (*P* = 0.002; *r*^2^ = 0.51) (**Figure 4**). From the 31 C-sources, 11 were significantly more utilized by the rhizosphere community while 4 were more utilized by the bulk soil community (*P* < 0.05). The performances of the communities on individual carbon sources were used to calculate the community niche of bulk soil and rhizosphere samples, and were significantly higher (*P* < 0.05) in the rhizosphere (29.03 ± 2.90) compared to bulk soil (23.57 ± 0.30). The most catabolized C-sources by the microbial community from rhizosphere (*P* < 0.05) were D-galacturonic acid, D-galactonic acid, D-glucosaminic acid, L-asparagine, 4-hydroxybenzoic acid, D,L,a-glycerolphosphate, L-phenylalanine, L-threonine, L-serine, tween-40 and putrescine. On the other hand, A,D-lactose, D-xylose, *i*-erythritol and *y*-hydroxybutyric acid were the most consumed C-sources by the microbial community from the bulk soil (*P* < 0.05) (**Figure 4**).

### Metagenome Prediction of Bulk Soil and Rhizosphere Bacterial Communities

**FIGURE 5:**
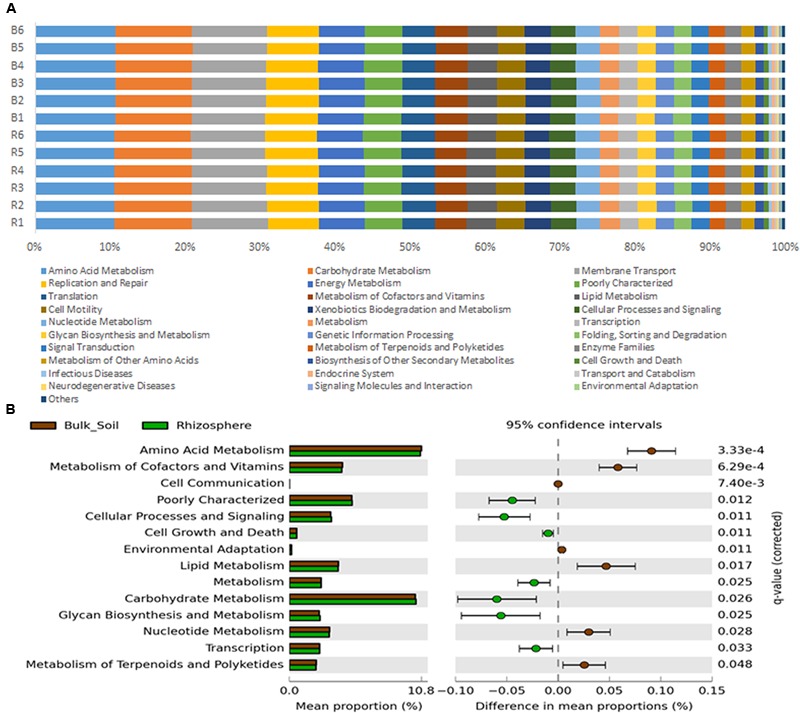
**(A)** Metagenome predicted functions classified using KEGG level 2 database in PICRUSt software, showing the most abundant functions throughout the 12 samples. **(B)** Statistical comparison (Welch’s *t*-test) between the predicted functions abundance on rhizosphere and bulk soil using the Benjamini–Hochberg *P*-value correction (*P* < 0.05).

Metagenomes were predicted from the 16S rRNA gene sequences with an accuracy based on a NSTI average value of 0.23 ± 0.02, which is typical for soil samples analyzed in other studies (Zarraonaindia et al., 2015; Chen et al., 2016). Despite such a high NSTI value, Zarraonaindia et al. (2015) found a significant similarity between the sequenced and predicted metagenome. Metagenome prediction resulted in more than 6,900 protein-coding genes, of which 153 were differentially predicted in accordance to their frequencies in bulk soil or rhizosphere (*P* < 0.05); 80 were significantly more abundant in the rhizosphere and 73 were significantly higher in bulk soil (**Supplementary Figure S1**). The predicted protein-coding genes were categorized by function using the KEGG level 2 GO, revealing the prevalence (∼50%) of genes related to amino acid metabolism, carbohydrate metabolism, membrane transport, replication and repair and energy metabolism, in both bulk soil and rhizosphere samples (**Figure 5A**). The other half of the predicted functions were affiliated with more than 25 other functional categories. A Welch’s t-test indicated that rhizosphere and bulk soil were enriched in 6 and 7 functional categories (KEGG level 2), respectively (*P* < 0.05). More specifically, predicted genes more abundant in the rhizosphere were mainly associated with Cellular Processes and Signaling, Cell Growth and Death, Carbohydrate Metabolism, Metabolism, Glycan Biosynthesis and Metabolism and Transcription (**Figure 5B**). On the other hand, genes related to Metabolism of Amino Acids, Lipids, Nucleotide, Terpenoids and Polyketides, and Cell Communication and Environmental Adaptation were more abundant in the bulk soil (**Figure 5B**).

**FIGURE 6:**
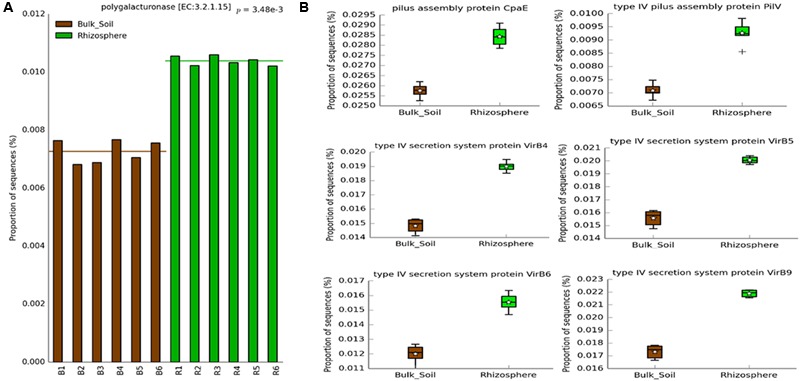
**Predicted genes on PICRUSt related to **(A)** CLPP analysis and **(B)** HGT, which showed significantly higher abundance in the rhizosphere (Welch’s *t*-test, *P* < 0.05) using the Bonferroni *P*-value correction**.

We observed a significantly higher abundance of predicted genes associated with HGT in the rhizosphere (**Figure 6B**) than in bulk soil. We found a higher prevalence of genes associated with conjugation, such as the pilus assembly protein CpaE and type IV pilus assembly protein PilV (Cabezón et al., 2015); as well as genes involved in transformation/conjugation, such as the type IV secretion system proteins VirB4, VirB5, VirB6, and VirB9 (**Figure 6B**) (Christie et al., 2005; Cabezón et al., 2015; Juhas, 2015). We also observed a greater number of predicted genes indirectly related to transduction, like DNA polymerase bacteriophage-type. Another interesting predicted gene, also more abundant in the rhizosphere, was related to colanic acid biosynthesis protein WcaH, which is associated with biofilm formation. To validate the results from the PICRUSt analysis, we looked for predicted genes coding for enzymes involved in the metabolism of the carbon sources present in the Biolog plate assays. We found a predicted gene associated with polygalacturonase, which was more abundant in the rhizosphere (**Figure 6A**). Likewise, the rhizosphere samples had higher degradation levels of D-galacturonic acid in the Biolog assays (**Figure 4**).

## Discussion

This study accessed the bacterial communities present in the sugarcane rhizosphere and bulk soil using real-time PCR and high-throughput sequencing in order to identify differences in bacterial abundance, diversity and taxonomic structure. Furthermore, the metabolic profile of the microbial communities was also assessed using CLPP and metagenome prediction with the aim of identifying potential traits important for the rhizosphere colonization.

Results of bacterial quantification (based on the quantity of 16S rRNA gene copies) provided the first indication of growth stimulus in the roots surroundings in our study, corroborating other studies (Wessén et al., 2010; Rachid et al., 2013). Differences in the bacterial community structure and composition between rhizosphere and bulk soil support the hypothesis that the rhizosphere selects a bacterial community from a pool in the bulk soil, which changes the abundance of specific groups according to their ability to adapt to the rhizosphere conditions. In this line, the similar values of richness and α-diversity found in these two environments provide supporting evidences that the rhizosphere shapes the bacterial community by modulating its composition (Mendes L.W. et al., 2014). Such distinction is better observed in the community structure based on the analysis of OTUs, where samples from each niche are separately plotted in the NMDS indicating a high β-diversity between rhizosphere and bulk soil.

Despite the fact that the bacterial communities were very different considering the analysis of OTUs, small changes were observed at the phyla level, where the taxonomic profile is quite similar (**Figure 1A**), suggesting that the shifts in abundances of specific groups are not phylogenetically dramatic. Three phyla, Acidobacteria, Proteobacteria and Actinobacteria were the most abundant in our study, similarly to those observed by Rachid et al. (2013), who analyzed bacterial communities of sugarcane fields under two different managements. In our study, from these 3 phyla only Actinobacteria was present at relatively higher abundance in bulk soil. Members of this phylum are known for their resistance to stress conditions (particularly drought stress) and/or ability to degrade complex carbon sources, explaining their higher abundance in the oligotrophic bulk soil environment (DeBruyn et al., 2011; Roes-Hill et al., 2011; Thomas et al., 2011; Kavamura et al., 2013). On the other hand, less abundant phyla, such as Nitrospirae, Verrucomicrobia, and Tenericutes, were enriched in the rhizosphere. Nunes da Rocha et al. (2013) showed that groups affiliated to Verrucomicrobia might be important to the rhizosphere of several plants, such as grasses, leek, and potatoes. Similarly, Verrucomicrobia and Nitrospirae were also detected with significant higher abundance in the rhizosphere of oats (DeAngelis et al., 2009) and soybean (Mendes L.W. et al., 2014). This evidence suggests that although understudied, these phyla can be commonly selected by the roots of several different plants and might play a critical role for their development. Proteobacteria is another bacterial phylum known for colonizing the rhizosphere (Uroz et al., 2010; Peiffer et al., 2013). Here we did not find differences when compared the abundance of members of this phylum in each niche. However, we found relatively higher abundances of families such as Burkholderiaceae, Pseudomonadaceae and Rhizobiaceae, which belong to Proteobacteria and are widely described as plant growth promoters (García-Fraile et al., 2012; Suárez-Moreno et al., 2012; Redondo-Nieto et al., 2013), in the rhizophere (**Figure 3**). These observations indicate the need to compare these communities in more detailed taxonomy levels than phyla, where they seem more similar.

Community level physiological profile results provide the first evidence of how the plant roots select microbes for its rhizosphere microbiome. PCA based on C-sources oxidation showed that the rhizosphere samples were closer to each other, while the bulk soil samples were more dispersed in the chart, suggesting that the microbial communities from the rhizosphere share a very similar functional community structure (**Figure 4**). This suggests that the rhizosphere selection effect can be possibly more effective for functional attributes than for taxonomy – as observed in the ordination of samples based on OTUs (**Figure 1B**).

We could conclude from both the number of carbon sources consumed in each habitat and the community niche analysis that the rhizosphere microbial community is more metabolically active. It might be that cells in an active state preferentially colonize the rhizosphere microbiome, as the roots are constantly supplying nutrients to this niche. In counterpart, the bulk soil community is more prone to undergo long periods without labile carbon, which might decrease the metabolic rate of most microbes. Our results also indicate that the labile C-sources released in the rhizosphere might be the main plant compounds acting in the microbiome selection. This is supported by the preferential use of the D-galacturonic acid, and its reduced form D-galactonic acid, in the rhizosphere.D-galacturonic acid is the most abundant component of pectin (Zhang et al., 2011), a major constituent of plant cell walls, which is consequently released in the rhizosphere. D-galacturonic acid has also been found as a component of root exudates (Tawaraya et al., 2015). It is important to note that CLPP is culture dependent, meaning that the measurements of C-sources oxidation reflect the activity of only part of the community in the sample, and is therefore limited in its ability to provide the real functional capacity of a microbial community *in vivo*.

The only common plant-produced C-source that was more utilized by the bulk soil bacterial community was D-xylose, a component of hemicellulose. Nevertheless, hemicellulose is one of the polysaccharide constituents of lignocellulose, the most recalcitrant component of the plant cell wall, which is only degraded by specialized enzymes (Mansour et al., 2016). In sugarcane field soils, the presence of hemicellulose is even higher, as leaves are constantly falling and accumulating on the soil surface (Chandel et al., 2012).

Although evidence for selection in the rhizosphere is clear, the bacterial abilities that enable rhizosphere colonization are still poorly described. To gain more insights on these abilities, PICRUSt was used to predict the metagenome of each of the samples. PICRUSt is dependent on available sequenced genomes and some bias can be produced if references for the species inhabiting the sampled environment are missing. However, it is a useful tool to provide insights about the community functional potential in the absence of shotgun metagenomic data. The combination of metagenome prediction and CLPP provided insights into community functional structure and reduced the bias related to each approach. For both communities, rhizosphere and bulk soil, the most prevalent predicted functions were Amino Acid Metabolism and Carbohydrate Metabolism, corroborating a previous study of sugarcane rhizosphere communities by metaproteomics (Lin et al., 2013). Some significant differences between bulk soil and rhizosphere were found, but the overall functional profiles classified by KEGG (level 2) were very similar, meaning that all higher functions are probably executed in both environments. Functions related to basic metabolism were found in higher abundance in the rhizosphere compared to the bulk soil (Cell Growth and Death, Metabolism, Carbohydrate Metabolism, Glycan Biosynthesis and Transcription), what can be linked to the higher metabolic activity in the rhizosphere communities detected on the CLPP analysis. These convergent results also suggest that the fast growth rate observed in previous studies of single species can be a widespread feature for rhizosphere colonization (Simons et al., 1996; Tan et al., 2013). In contrast, the bulk soil community showed a higher ratio of functions related to secondary metabolism, including the degradation of complex compounds and environmental resistance (Metabolism of Terpenoids and Polyketides, and Environmental Adaptation).

A deeper investigation of the metagenome prediction showed a greater abundance of genes related to Colanic Acid Biosynthesis Protein WcaH in the rhizosphere. Colanic acid is associated with biofilm formation in *Escherichia coli* (Danese et al., 2000), which is in agreement with previous data that showed the importance of biofilm formation for colonization of the roots surface (Ramey et al., 2004; Rinaudi and Giordano, 2010; Tan et al., 2013). Another interesting result obtained from the metagenome prediction was the higher abundance of genes related to HGT in the rhizosphere. We found genes related to bacterial transformation and conjugation, such as those associated with the Type IV Secretion System, enriched in the rhizosphere (Christie et al., 2005; Cabezón et al., 2015; Juhas, 2015). We also found a higher abundance of genes related to conjugation, as indicated by the occurrence of genes codifying the pilus assembly protein CpaE and type IV pilus assembly protein PilV (Cabezón et al., 2015); and genes possibly related to transduction, such as genes related to bacteriophages. These results suggest that HGT could be an important mechanism for bacterial adaptation in the rhizosphere. Given that the accumulation of beneficial mutations is a slow process to generate genetic variability in the short time scale (Gogarten et al., 2002; Jain et al., 2003), the horizontal transfer of entire genes may be a more efficient way for bacteria to rapidly adapt to the rhizosphere niche. This process is widely understood as a powerful tool for the rapid evolution of antibiotic resistance in bacteria inhabiting hospital environments (Palmer and Kishony, 2013), and may happen in an analogous way for rhizosphere colonization.

Although there is some bias in using 16S rRNA gene libraries to predict metagenomes, other studies based on real metagenome sequencing found results consistent with and supporting our data. Mendes L.W. et al. (2014) found an enrichment of Type IV Secretion System genes in the rhizosphere of soybean. Similarly, Alzubaidy et al. (2016) found a significant higher number of genes linked to Phages, Prophages, Transposable Elements, and Plasmids in the rhizosphere of the mangrove plant *Avicennia marina*. Together with our results, this evidence suggests that HGT may be a widespread mechanism that facilitates rhizosphere colonization in plants.

Moreover, to support the results of the functional analyzes, we checked for matches between the C-sources degradation in the CLPP analysis and the enzyme-coding genes predicted by PICRUSt. Even considering the bias in each approach, a match was observed for genes related to polygalacturonase, more abundant in the rhizosphere samples, and the preferential degradation of D-galacturonic acid by the rhizospheric microbial community. This meaningful finding indicates that the capacity to metabolize the galacturonic acid may be an important trait for rhizosphere colonization. Other genes related to the degradation of specific CLPP C-sources were found in the metagenome prediction, but no significant differences were observed between rhizosphere and bulk soil. The lack of other matches might be related to the fact that CLPP measures differences in gene content as well as differential expression of genes, while metagenome prediction is based exclusively on the prediction of genotypes. It might also be that the low frequency of groups hosting these genes limited its prediction in the PICRUSt approach, or even that this approach may not have the accuracy to properly detect small changes in gene abundance of functional groups. The high NSTI values found in our samples and the lack of other approach to confirm the prediction also tell that the finding of HGT-related genes higher in the rhizosphere is an interesting suggestion that this activity can be enriched in this environment, but we highlight the need to experimentally confirm this issue in next studies.

Taken together, our results show important findings concerning the taxonomic and functional selection exerted by the sugarcane rhizosphere on the bacterial community, as well as identified potential traits that allow bacteria to colonize the rhizosphere environment. We firstly highlight the ability to degrade the D-galacturonic acid, a compound potentially used by the plant in a biochemical selection; and secondly the potential ability to perform HGT, which can spread important genes related to rhizosphere colonization, providing the bacteria an evolutionary and ecological advantage.

## Author Contributions

LL performed the molecular, bioinformatics and statistical analyzes, designed the experiment, interpreted the data and wrote the manuscript; MdS performed the Biolog and bioinformatics analyzes, designed the experiment, interpreted the data and wrote the manuscript; FA contributed to the conception of the work and design of the experiment, interpreted the data and wrote the manuscript.

## Conflict of Interest Statement

The authors declare that the research was conducted in the absence of any commercial or financial relationships that could be construed as a potential conflict of interest.

## Supplementary Material

FIGURE S1 **Statistical comparison (Welch’s *t*-test) between the predicted genes abundance on rhizosphere and bulk soil samples using the Bonferroni *P*-value correction (*P* < 0.05)**.
