# Supplementary figures and images for: Bacterial Abilities and Adaptation Toward the Rhizosphere Colonization
Source: Front Microbiol. 2016 Aug 25;7:1341. doi: 10.3389/fmicb.2016.01341 (PMC4997060; doi:10.3389/fmicb.2016.01341)

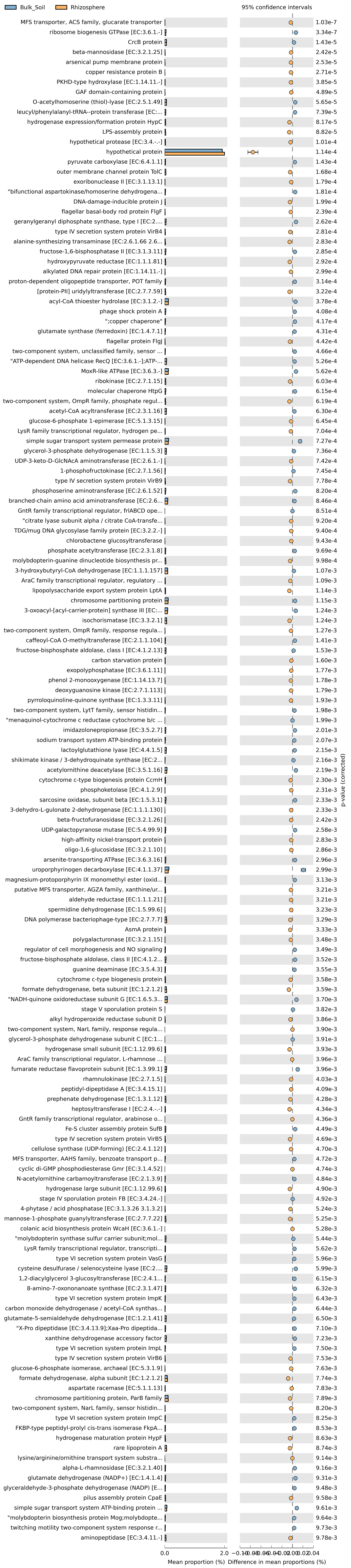

Supplement: FIGURE S1 — Statistical comparison (Welch’s t-test) between the predicted genes abundance on rhizosphere and bulk soil samples using the Bonferroni P-value correction (P < 0.05). [file Image_1.TIF]
