# Supplementary material for: Bacterial Abilities and Adaptation Toward the Rhizosphere Colonization
Source: Front Microbiol. 2016 Aug 25;7:1341. doi: 10.3389/fmicb.2016.01341 (PMC4997060; doi:10.3389/fmicb.2016.01341)
Supplement: Supplementary file 2 [file Table_1.DOCX]

**Table S1.** Primers used in the second reaction for the Illumina sequencing

| 16SV4FPCR1_1  F | 5’ TCGTCGGCAGCGTCAGATGTGTATAAGAGACAGAYTGGGYDTAAAGNG |
| --- | --- |
| 16SV4FPCR1_2 F | 5’ TCGTCGGCAGCGTCAGATGTGTATAAGAGACAGNAYTGGGYDTAAAGNG |
| 16SV4FPCR1_3 F | 5’ TCGTCGGCAGCGTCAGATGTGTATAAGAGACAGNNAYTGGGYDTAAAGNG |
| 16SV4FPCR1_4 F | 5’ TCGTCGGCAGCGTCAGATGTGTATAAGAGACAGNNNAYTGGGYDTAAAGNG |
|  |  |
| 16SV4RPCR1_1 R | 5’ GTCTCGTGGGCTCGGAGATGTGTATAAGAGACAGCCGTCAATTCMTTTRAGT |
| 16SV4RPCR1_2 R | 5’ GTCTCGTGGGCTCGGAGATGTGTATAAGAGACAGNCCGTCAATTCMTTTRAGT |
| 16SV4RPCR1_3 R | 5’ GTCTCGTGGGCTCGGAGATGTGTATAAGAGACAGNNCCGTCAATTCMTTTRAGT |
| 16SV4RPCR1_4 R | 5’ GTCTCGTGGGCTCGGAGATGTGTATAAGAGACAGNNNCCGTCAATTCMTTTRAGT |
